# The impact of revised diagnostic criteria on hospital trends in gestational diabetes mellitus rates in a high income country

**DOI:** 10.1186/s12913-020-05655-y

**Published:** 2020-08-26

**Authors:** Léan E. McMahon, Eimer G. O’Malley, Ciara M. E. Reynolds, Michael J. Turner

**Affiliations:** 1 National Women and Infants Health Programme, Coombe Women and Infants University Hospital, Dublin, Ireland; 2 UCD Centre for Human Reproduction at Coombe Women and Infants University Hospital, Cork Street, Dublin 8, Ireland

**Keywords:** Gestational diabetes mellitus, Oral glucose tolerance test, Selective testing, Pregnancy risk factors

## Abstract

**Objective:**

In 2010, national guidelines were published in Ireland recommending more sensitive criteria for the diagnosis of Gestational Diabetes Mellitus (GDM). The criteria were based on the 2008 Hyperglycemia and Adverse Pregnancy Outcomes (HAPO) Study and were endorsed subsequently by the World Health Organization (WHO). Screening nationally is selective based on risk factors. We examined the impact of the new criteria on hospital trends nationally for GDM over the 10 years 2008–17.

**Research design and methods:**

Data from three national databases, the Hospital Inpatient Enquiry System (HIPE), National Perinatal Reporting System (NPRS) and the Irish Maternity Indicator System (IMIS), were analyzed using descriptive statistics, analysis of variance, and Poisson loglinear modelling.

**Results:**

The overall incidence of GDM nationally increased almost five-fold from 3.1% in 2008 to 14.8% in 2017 (*p* ≤ 0.001). The incidence varied widely across maternity units. In 2008, the incidence varied from 0.4 to 5.9% and in 2017 it varied from 1.9 to 29.4%. There were increased obstetric interventions among women with GDM over the decade, specifically women with GDM having increased cesarean sections (CS) and induction of labor (IOL) (*p* ≤ 0.001). These trends were significant in large and mid-sized maternity hospitals (*p* ≤ 0.001). The increase in GDM diagnosis could not be explained by an increase in maternal age nationally over the decade. The data did not include information on other risk factors such as obesity. The increased incidence in GDM diagnosis was accompanied by a decrease in high birthweight ≥ 4.5 kg nationally.

**Conclusions:**

We found adoption of the new criteria for diagnosis of GDM resulted in a major increase in the incidence of GDM rates. Inter-hospital variations increased over the decade, which may be explained by variations in the implementation of the new national guidelines in different maternity units. It is likely to escalate further as compliance with national guidelines improves at all maternity hospitals, with implications for provision and configuration of maternity services. We observed trends that may indicate improvements for women and their offspring, but more research is required to understand patterns of guideline implementation across hospitals and to demonstrate how increased GDM diagnosis will improve clinical outcomes.

## Introduction

There is a lack of consensus worldwide about screening and testing for Gestational Diabetes Mellitus (GDM). Screening may be universal or selective based on maternal or fetal risk factors. However, the risk factors applied may vary and adherence is rarely measured. Testing is usually with an Oral Glucose Tolerance Test (OGTT) at 24–28 weeks gestation but the glucose load administered may be 75 g or 100 g. Testing may be one-step or two-step where a 50 g Glucose Challenge Test preceded the 75 g or 100 g OGTT. Finally, different measurements of maternal plasma glucose may be used for diagnostic purposes.

Following publication of the Hyperglycemia and Adverse Pregnancy Outcomes (HAPO) study in 2008, the International Association of the Diabetes and Pregnancy Study Groups (IADPSG) recommended more sensitive criteria for diagnosing GDM [1, 2]. The new criteria have been controversial [3–8]. The treatment benefit for additional women diagnosed by the new criteria is unclear and, furthermore, the new diagnostic thresholds have significant impact on costs and on infrastructure capacity [9]. Both the screening and diagnostic criteria vary among countries and commonly between obstetric and endocrinology organizations in a single country. For example, in the United States of America (USA), the new criteria were initially recommended by the American Diabetes Association (ADA), but not the American College of Obstetricians and Gynecologists (ACOG), which recommends the two-step screening approach using the Carpenter and Coustan or the National Diabetes Data Group criteria [10, 11]. In the interests of consensus, the World Health Organization (WHO) endorsed the IADPSG criteria in 2013 [12]. While the 2013 WHO criteria are becoming more widely accepted and used, the issue continues to be debated among the main diabetes and obstetric organisations.

Like most European countries, in Ireland women are screened for GDM selectively based on maternal and fetal risk factors. There is no universal screening in Ireland. Up until 2011, the diagnosis was based on the 100 g OGTT and the Carpenter Coustan diagnostic criteria were applied. The first national guideline on GDM published in August 2010 recommended that women be tested with a 75 g OGTT and that the IADPSG/WHO diagnostic criteria be applied [13]. The new criteria reduced the abnormal fasting plasma glucose level from 5.3 to 5.1 mmol/l, the 1-h remained unchanged, the abnormal 2-h was reduced from 8.6 to 8.5 mmol/l, and the 3-h measurement was omitted. Importantly, the diagnosis required only one, not two, abnormal values.

Our group previously examined the impact of these new criteria on testing for GDM in our own hospital. We found the introduction of the new criteria in January 2011 resulted in a 59% increase of GDM diagnoses in the first year from 139 to 221 cases (*p* = 0.02) [14]. The increase was mainly due to the larger numbers of women who had increased fasting plasma glucose (FPG), as well as improved compliance with selective screening.

A randomized controlled trial showed that universal screening using the IADPSG criteria may increase GDM diagnosis by up to 15–20% of the obstetric population [15]. This raises questions regarding increased healthcare practices and costs for both women and service providers. It has been estimated that the new criteria will impact on the workload for diabetes care teams by 22–32% [16]. Potentially, it may also lead to an increase in interventions such as induction of labor (IOL) and Cesarean section (CS) [17]. More sensitive criteria may also increase the psychological burden for some women during pregnancy [18].

The aim of this study was to examine national and hospital trends of the revised, more sensitive diagnostic criteria for GDM in women tested selectively using the one-step 75 g OGTT. In view of the association reported in the HAPO study between GDM and large for gestational age (LGA) infants, we also examined the impact of the new criteria on birth weight over the same decade.

## Research design and methods

### Background

In the Republic of Ireland, maternity services are predominantly hospital-based, with approximately 0.3% of births occurring at home [19]. There are 19 publicly funded maternity hospitals that admit women without differentiation from all socioeconomic groups, irrespective of whether or not they have private health insurance. The hospitals provide public and private maternity care. They vary in size, based on the number of births per annum. In 2017, for example, the four largest university hospitals had over 7000 births each and the smallest unit had 980 births [20]. Since 2014, there have been no exclusively private maternity hospitals nationally.

The Republic of Ireland incorporated the IADPSG criteria into our revised national guidelines published in August 2010. The guidelines recommend screening women selectively for GDM with a one-step 75 g OGTT based on risk factors [13, 21]. Screening was recommended at 24–28 weeks gestation, but screening based on factors such as polyhydramnios or fetal macrosomia may not take place until the third trimester. Ireland’s maternity hospitals varied in the extent and timing of their adoption of the new IADPSG recommendations [22].

### Data sources

Data were obtained from several national sources. 1) We obtained numerator data for GDM from the Hospital In-Patient Enquiry (HIPE) system. Established in 1971, the HIPE is a health information system designed to collect clinical and administrative data on discharges from, and deaths in, acute hospitals in Ireland. The source document for HIPE coding is the medical record or chart. The data are coded according to the International Statistical Classification of Diseases and Related Health Problems, Tenth Revision, Australian Modification (ICD-10-AM). The HIPE system is managed by the Healthcare Pricing Office (HPO). We extracted HIPE data for each year from 2008 through 2017 from the 19 matenrity hospitals/units. The selection was All diagnoses (ICD-10-AM) equal to o244 (Diabetes Mellitus arising during pregnancy), including inpatients and day-cases. The individual hospital reports provided total numbers of discharges for women with GDM attending public and private maternity care.

Data for CS and IOL were also obtained from the HIPE. Data for CS were based on the selection for All Procedures (ICD-10-AM Block) equal to 1340 (Cesarean section) and data for IOL were based on the selection for All Procedures (ICD-10-AM Block) equal to 1334 (Medical or surgical induction of labor). Hospital reports for both these selections were developed for individual years from 2008 through 2017.

2) Denominator data for numbers of mothers delivered per hospital per annum were obtained from the National Perinatal Reporting System (NPRS) (2008–16) and the Irish Maternity Indicator System (IMIS) [20, 23]. Data for birthweight were obtained from the NPRS. The most recent data available were for 2016. The NPRS is derived from the Birth Notification Form (BNF01), where all births are notified and registered nationally. The IMIS is the national management instrument that facilitates monthly and annual monitoring of data for selected key metrics at the 19 maternity hospitals (e.g., demographics, deliveries, obstetric risks/complications, neonatal metrics, and breastfeeding).

### Data analysis

The data were prepared and charts were compiled using MS Excel. Data were analyzed using the Statistical Programme for Social Sciences (SPSS). Normality of continuous data was assessed using descriptive statistics for extreme values, missing cases, skewness and kurtosis, and visual inspection of histograms and Q-Q Plots. The data were positively skewed and leptokurtic, as may be expected, due to the large numbers of deliveries concentrated at four large maternity hospitals and the peaked frequencies of relatively small values. There were no missing hospital data.

The data were analyzed using ANOVA models including total mothers delivered, total CS, total IOL, total GDM, women’s average age (all interval variables), and hospital size (nominal variable). Statistics for analysis of variance were obtained using the Welch test for equality of means. We used Poisson loglinear models to model the numbers of total GDM and women with GDM who had CS and IOL. National rates of stillbirth, birthweight ≥4.5 kg, and birthweight below 2.5 kg were examined separately.

National data on risk factors such as maternal obesity or family history were not available. Compliance with the implementation in individual hospitals of selective screening for GDM based on risk factors is not audited nationally and, therefore, was not incorporated in the data analysis.

## Results

**Fig. 1:**
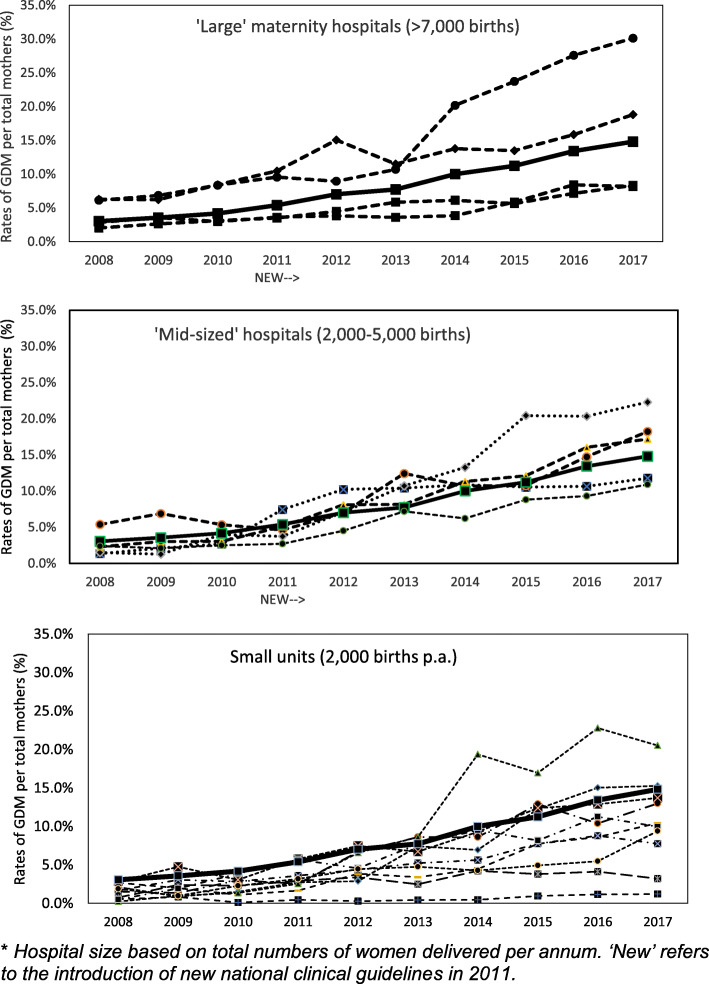
Incidence of GDM nationally and at the 19 maternity hospitals*. * *Hospital size based on total numbers of women delivered* per annum*. ‘New’ refers to the introduction of new national clinical guidelines in 2011. (Sources: Hospital In-Patient Enquiry System (HIPE) 2008–2017, National Perinatal Reporting System (NPRS) 2008–2013, Irish Maternity Indicator System (IMIS) 2014–2017)*

Over the decade 2008–17, the incidence of GDM diagnosis (based on hospital discharges) increased from 3.1% (95% CI 2.9–3.2) to 14.8% (95% CI 14.5–15.1) of mothers delivered (*p* ≤ 0.001). The incidence of GDM was relatively consistent prior to the introduction of the IADPSG criteria, with national rates ranging from 3.1% in 2008 to 4.2% in 2010 (*p* = 0.018). The variation started to become more pronounced after the introduction of the new IADPSG diagnostic criteria in 2011 and particularly so after around 2014. There was a 93.3% change from 5.4% in 2011 to 14.8% in 2017 (*p* ≤ 0.001) (Fig. 1).

Descriptive statistics show significant changes in total GDM over the decade across all hospitals and in hospitals/units of different sizes. They also show significant changes in total GDM (*p* = 0.021) and in women with GDM having CS and IOL (*p* ≤ 0.001) (Table 1).

**Table 1:** Univariate descriptive statistics of all maternity hospitals/units in Ireland (large, mid-sized, small)^a^ and tests of variance, 2008–17

			Median	Mean	S.D.	95% CI		Min	Max	F(Sig.)	Welch
All hospitals (*n* = 19)	Total GDM	2008	45.0	116.6	159.0	40.0-	193.2	5	527	2.222*	.021
		2009	51.0	137.4	175.4	52.9-	221.9	16	598		
		2010	60.0	160.2	216.1	56.0-	264.3	2	738		
		2011	71.0	203.3	261.8	77.1-	329.5	9	894		
		2012	110.0	255.8	311.7	105.6-	406.0	5	1267		
		2013	166.0	271.5	268.0	142.3-	400.7	8	926		
		2014	172.0	347.0	436.3	136.7-	557.3	8	1773		
		2015	227.0	381.0	462.1	158.3-	603.7	16	1983		
		2016	267.0	443.0	546.0	179.8-	706.2	19	2319		
		2017	251.0	473.6	592.5	188.0-	759.1	19	2479		
		Total	568.0	278.9	384.0	224.0-	333.9	2	2479		
	CS and GDM	2008	11.0	25.8	29.7	11.6-	40.1	0	98	3.708***	.000
		2009	17.0	31.3	31.0	16.3-	46.2	3	111		
		2010	22.0	34.6	36.6	17.0-	52.2	1	130		
		2011	22.0	47.9	51.6	23.0-	72.8	1	181		
		2012	29.0	58.5	61.4	28.9-	88.1	1	245		
		2013	44.0	66.7	62.2	36.7-	96.7	2	234		
		2014	50.0	77.7	75.4	41.4-	114.1	3	283		
		2015	58.0	84.3	73.7	48.8-	119.8	2	258		
		2016	63.0	103.2	92.9	58.4-	147.9	3	339		
		2017	57.0	107.8	104.3	57.6-	158.1	3	359		
		Total	140.0	63.8	70.6	53.7-	73.9	0	359		
	IOL and GDM	2008	10.0	20.7	26.4	8.0-	33.5	0	84	4.007***	.000
		2009	10.0	28.1	33.2	12.1-	44.1	3	106		
		2010	18.0	34.1	38.5	15.5-	52.6	1	134		
		2011	25.0	45.4	50.8	20.9-	69.9	0	176		
		2012	36.0	58.3	64.8	27.0-	89.5	0	242		
		2013	42.0	66.3	72.9	31.2-	101.5	2	278		
		2014	51.0	79.9	78.8	42.0-	117.9	0	248		
		2015	56.0	84.6	71.5	50.1-	119.0	4	248		
		2016	73.0	102.9	93.6	57.9-	148.1	3	311		
		2017	64.0	110.8	105.1	60.2-	161.5	2	402		
		Total	135.5	63.1	72.9	52.7-	73.6	0	402		
Large hospitals (*n* = 4)	Total GDM	2008	389.0	370.0	181.0	82.1-	658.0	527	175	1.552 (ns)	.366
		2009	433.0	424.3	172.2	150.2	698.3	598	233		
		2010	505.5	505.3	258.6	83.8-	916.7	738	272		
		2011	603.0	600.3	326.3	81.0-	1119.5	894	301		
		2012	583.5	694.5	432.9	5.6-	1383.3	1267	344		
		2013	698.0	659.5	310.7	165.1-	1153.9	926	316		
		2014	836.0	949.3	660.8	–	–	1773	352		
		2015	823.0	1019.0	706.1	–	–	1983	447		
		2016	1024.5	1225.0	798.7	–	–	2319	532		
		2017	1094.5	1317.8	873.6	–	–	2479	603		
		Total	568.0	776.5	567.4	595.0-	958.0	2479	175		
	CS and GDM	2008	77.0	70.5	29.9	22.9-	118.1	98	30	4.481*	.016
		2009	82.0	82.3	24.1	43.9-	120.6	111	53		
		2010	96.5	91.5	39.0	29.4-	153.6	130	43		
		2011	125.0	121.0	58.6	27.7-	214.3	181	53		
		2012	117.5	140.5	72.2	25.5-	255.5	245	82		
		2013	161.0	158.5	64.3	56.2-	260.8	234	78		
		2014	198.0	191.3	84.4	56.9-	325.6	283	86		
		2015	203.0	198.8	68.2	90.2-	307.3	258	131		
		2016	241.5	248.8	81.1	119.8-	377.7	339	173		
		2017	275.0	271.8	94.5	121.4-	422.1	359	178		
		Total	140.0	157.5	88.1	129.3-	185.7	359	30		
	IOL and GDM	2008	70.0	66.3	21.0	32.9-	99.6	84	41	4.776**	.004
		2009	88.0	82.8	25.67	41.9-	123.6	106	49		
		2010	104.0	96.0	38.68	34.5-	157.6	134	42		
		2011	122.5	119.8	49.71	40.7-	198.9	176	58		
		2012	124.5	136.8	80.0	9.5-	264.0	242	56		
		2013	162.5	161.3	101.1	0.4-	322.1	278	42		
		2014	240.5	203.8	78.8	78.3-	329.2	248	86		
		2015	212.5	198.5	54.7	111.5-	285.5	248	121		
		2016	274.5	257.3	67.1	150.4-	364.1	311	169		
		2017	276.0	280.3	97.9	124.5-	436.0	402	167		
		Total	135.5	160.3	91.7	130.9-	189.6	402	41		
Mid-sized units (*n* = 5)	Total GDM	2008	73.0	94.0	59.3	20.3-	167.7	194	43	6.180**	.001
		2009	113.0	112.8	79.2	14.4-	211.2	238	37		
		2010	121.0	124.6	45.0	68.8-	180.4	185	60		
		2011	155.0	178.2	121.7	27.1-	329.3	378	65		
		2012	231.0	261.6	147.6	78.3-	444.9	494	100		
		2013	293.0	314.4	117.2	168.9-	459.9	473	166		
		2014	315.0	321.4	127.0	163.7-	479.1	476	128		
		2015	392.0	352.6	123.4	209.3-	515.9	487	175		
		2016	434.0	400.8	129.5	240.0-	561.6	503	176		
		2017	510.0	440.0	137.5	269.2-	610.8	519	197		
		Total	214.0	261.0	158.8	215.9-	306.2	519	37		
	CS and GDM	2008	16.0	19.4	19.4	−4.6-	43.4	51	2	4.590**	.003
		2009	36.0	30.2	15.7	10.7-	49.7	46	7		
		2010	37.0	34.2	15.1	15.5-	52.9	48	10		
		2011	43.0	54.4	34.5	11.5-	97.3	105	20		
		2012	40.0	68.8	49.3	7.6-	130.0	140	24		
		2013	62.0	77.0	31.5	37.9-	116.1	127	53		
		2014	76.0	80.2	36.1	35.4-	125.0	134	35		
		2015	80.0	90.2	35.2	46.5-	133.9	146	58		
		2016	113.0	110.8	44.6	55.4-	166.2	154	48		
		2017	135.0	116.8	46.7	58.8-	174.8	173	53		
		Total	53.5	68.2	44.9	55.4-	81.0	173	2		
	IOL and GDM	2008	12.0	13.0	9.1	1.8-	24.2	24	2	5.213***	.000
		2009	31.0	27.4	16.9	6.5-	48.3	47	10		
		2010	28.0	32.0	13.2	15.6-	48.4	50	16		
		2011	27.0	44.6	40.5	−5.7-	94.9	117	24		
		2012	43.0	73.2	59.8	−1.1-	147.5	179	41		
		2013	67.0	77.8	44.0	23.2-	132.4	153	45		
		2014	72.0	77.8	37.3	31.5-	124.2	142	51		
		2015	91.0	89.6	31.5	50.5-	128.7	138	53		
		2016	94.0	107.0	30.5	69.2-	144.8	153	78		
		2017	111.0	115.0	24.2	84.9-	145.1	139	80		
		Total	51.5	65.7	45.4	52.9-	78.6	179	2		
Small units (*n* = 10)	Total GDM	2008	28.0	26.5	15.4	15.5-	37.5	50	5	6.521***	.000
		2009	31.5	35.0	16.6	23.2-	46.9	64	16		
		2010	42.0	39.9	21.8	24.3-	55.5	85	2		
		2011	57.0	57.1	29.9	35.7-	78.5	125	9		
		2012	75.5	77.4	34.5	52.7-	102.1	134	5		
		2013	82.0	94.8	52.5	57.3-	132.3	169	8		
		2014	89.5	118.9	92.4	52.8-	185.0	350	8		
		2015	126.0	135.0	75.5	81.0-	189.0	269	16		
		2016	134.5	151.3	94.4	83.8-	218.8	337	19		
		2017	156.5	152.7	86.3	91.0-	214.4	311	19		
		Total	67.0	88.9	74.0	74.2-	103.5	350	2		
	CS and GDM	2008	8.5	11.2	12.1	2.5-	19.9	43	0	7.146***	.000
		2009	11.5	11.4	4.8	7.9-	14.8	18	3		
		2010	10.0	12.0	7.7	6.5-	17.5	22	1		
		2011	19.0	15.4	8.6	9.2-	21.6	27	1		
		2012	22.0	20.6	8.5	14.5-	26.7	30	1		
		2013	23.0	24.8	12.9	15.6-	34.0	44	2		
		2014	30.5	31.1	16.7	19.1-	43.1	56	3		
		2015	39.5	35.5	16.1	24.0-	47.0	61	2		
		2016	41.5	41.1	21.4	25.8-	56.4	80	3		
		2017	38.5	37.8	18.8	24.3-	51.3	68	3		
		Total	21.0	24.1	17.2	20.7-	27.5	80	0		
	IOL and GDM	2008	5.0	6.4	5.5	2.5-	10.3	18	0	7.983***	.000
		2009	5.5	6.6	3.7	3.9-	9.3	12	3		
		2010	9.5	10.3	7.3	5.1-	15.6	25	1		
		2011	14.5	16.0	13.9	6.0-	26.0	45	0		
		2012	19.5	19.4	11.4	11.3-	27.5	36	0		
		2013	21.0	22.6	12.8	13.4-	31.8	48	2		
		2014	28.0	31.5	17.4	19.1-	43.9	65	0		
		2015	30.5	36.5	20.7	21.7-	51.3	71	4		
		2016	38.0	39.2	22.1	23.4-	55.0	73	3		
		2017	46.5	41.0	20.8	26.1-	55.9	64	2		
		Total	18.5	23.0	19.1	19.2-	26.78	73	0		

^a^Large hospitals: > 7000 deliveries per annum; Mid-sized hospitals/units: 2000–6000 deliveries p.a., Small units: < 2000 deliveries p.a. (based on numbers of deliveries in 2017)

In 2008, the inter-hospital rate of GDM ranged from 0.4% at a small unit to 5.9% at one of the largest maternity hospitals (difference of 14.7-fold). In 2017, inter-hospital rates varied from 1.9 to 29.4% (difference of 15.4). Among the four large maternity hospitals, GDM rates ranged from 4.3% in 2008 to 16.4% in 2017 (R^2^ = 0.95, *p* ≤ 0.01). Among the five mid-sized units, GDM rates ranged from 2.6% in 2008 to 16.1% in 2017 (R^2^ = 0.98, *p* ≤ 0.01). Among the ten smaller units, GDM rates ranged from 1.5% in 2008 to 10.6% in 2017 (R^2^ = 0.96, *p* ≤ 0.01).

Analysis of GDM rates over the years indicates significant variance according to hospital size. Large maternity hospitals had 8.7 times higher rates of GDM compared to smaller units and mid-size units had almost three times higher rates of GDM compared to smaller units (Table 2).

**Table 2:** Poisson log linear model showing estimates of total GDM and women with GDM having CS and IOL according to hospital size^a^, 2008–17

	Total GDM				Women with GDM & CS				Women with GDM & IOL			
	B	Sig.	Exp(B)	95% CI	B	Sig.	Exp(B)	95% CI	B	Sig.	Exp(B)	95% CI
Hospital size												
Large	2.17	.000	8.74	8.54–8.95	1.88	.000	6.53	6.23–6.85	1.94	.000	6.98	6.66–7.32
Mid-size	1.08	.000	2.94	2.86–3.02	1.04	.000	2.83	2.69–2.98	1.05	.000	2.86	2.72–3.02
Small (Ref)	–				–				–			
Year												
2008	−1.40	.000	0.25	0.24–0.26	−1.43	.000	0.24	0.22–0.26	−1.68	.000	0.19	0.17–0.21
2009	−1.24	.000	0.29	0.28–0.30	−1.24	.000	0.29	0.27–0.32	−1.37	.000	0.25	0.23–0.28
2010	−1.08	.000	0.34	0.33–0.35	−1.14	.000	0.32	0.29–0.35	−1.18	.000	0.31	0.28–0.34
2011	−0.85	.000	0.43	0.41–0.45	−0.81	.000	0.44	0.41–0.48	−0.89	.000	0.41	0.38–0.44
2012	−0.62	.000	0.54	0.52–0.56	−0.61	.000	0.54	0.50–0.58	−0.64	.000	0.53	0.49–0.57
2013	−0.56	.000	0.57	0.55–0.59	−0.48	.000	0.62	0.58–0.66	−0.54	.000	0.60	0.56–0.64
2014	−0.31	.000	0.73	0.71–0.76	−0.33	.000	0.72	0.67–0.77	−0.33	.000	0.72	0.68–0.77
2015	−0.22	.000	0.81	0.78–0.83	−0.25	.000	0.78	0.73–0.84	−0.27	.000	0.76	0.72–0.81
2016	−0.07	.000	0.94	0.91–0.96	−0.44	.160	0.96	0.90–1.02	−0.74	.190	0.93	0.87–1.00
2017 (Ref)	–				–				–			

^a^Large hospitals: > 7000 deliveries per annum; Mid-sized hospitals/units: 2000–6000 deliveries p.a., Small units: < 2000 deliveries p.a. (based on numbers of deliveries in 2017). Source: Hospital In-Patient Enquiry (HIPE) system 2008-2017

Large hospitals were not significantly different to each other (*p* = 0.175), but mid-sized and small maternity units were (*p* ≤ 0.001). The differences at these units were mostly observed in more recent years, for mid-sized units in 2016 (*p* = 0.046) and 2017 (*p* = 0.036) and for small units in 2012 (*p* = 0.023) and 2013 (*p* = 0.047) and from 2015 onwards (*p* = 0.026; 2016 *p* = 0.042, 2017 *p* = 0.023).

Table 2 shows significant variations in women with GDM having CS deliveries (*p* ≤ 0.001) and women with GDM having labor induced (*p* ≤ 0.001). The Poisson loglinear model estimating hospital size and change over the decade from 2008 to 2017 was statistically significant (*p* ≤ 0.001). The parameter estimates indicate that women with GDM having a CS are 6.5 times more likely in large maternity hospitals compared with small units (*p* ≤ 0.001) and 2.8 times more likely in mid-sized units (*p* ≤ 0.001). The likelihood of women with GDM having a CS was significantly increased over the years, from Exp(B) = 0.24 in 2008 to Exp(B) = 0.96 in 2016. Women with GDM were more likely to have a CS in 2017 compared to earlier years (*p* ≤ 0.001).

Similarly for women with GDM having labor induced. The parameter estimates indicate that women with GDM having IOL are 7.0 times more likely in large maternity hospitals compared with small units (*p* ≤ 0.001) and 2.9 times more likely in mid-sized units (*p* ≤ 0.001). The likelihood of women with GDM having IOL significantly increased over the years, from Exp(B) = 0.19 in 2008 to Exp(B) = 0.93 in 2016 showing women with GDM were increasingly more likely to have IOL as the decade progressed (*p* ≤ 0.001) (Table 2).

**Fig. 2:**
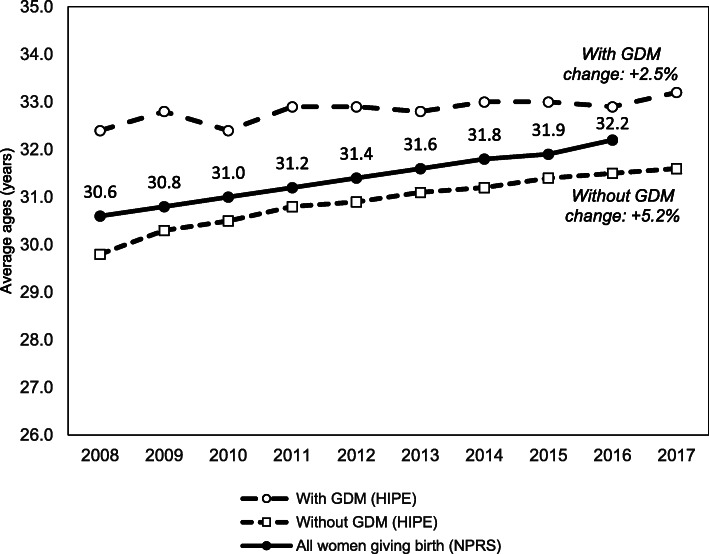
Average ages of all women giving birth (irrespective of parity) nationally and with/without GDM, 2008–2017. *(Sources: Hospital In-Patient Enquiry System (HIPE) 2008–2017, National Perinatal Reporting System (NPRS) 2008–2016)*

Women generally were older on average when giving birth in 2017 (mean = 31.7 years, S.D. = 0.8) compared with 2008 (mean = 29.8 years, S.D. = 0.8). Our study found women with GDM were older when giving birth (mean = 32.8 years, S.D. = 0.1), compared with women without GDM (mean = 30.9 years, S.D. = 0.06). The mean age of women with GDM was not significantly different in 2017 than 2008 (*p* = 0.353), whereas the mean age of women without GDM giving birth increased from 29.8 years in 2008 to 31.6 years in 2017 (*p* ≤ 0.001) (Fig. 2).

**Fig. 3:**
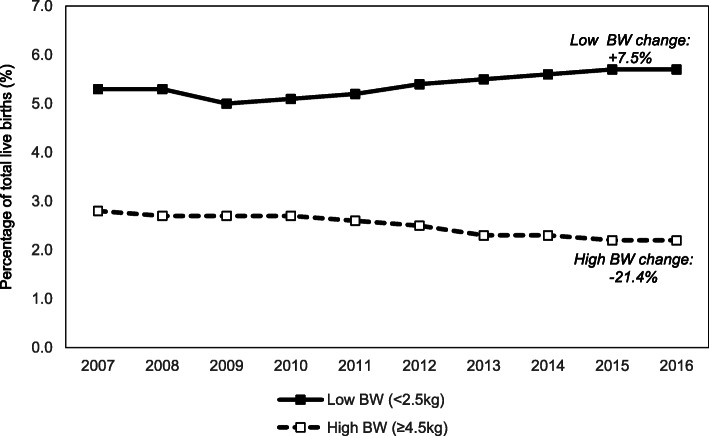
Percentages of total live births with low (<2.5 kg) and high (≥4.5 kg) birthweight (BW), 2007–2016. *(Source: National Perinatal Reporting System (NPRS) 2018)*

Over nine years from 2008 to 2016, the national average rate of high birthweight fell from 2.7% in 2008 to 2.2% in 2016 (R^2^ = 0.93, *p* ≤ 0.01). The national average rate of low birthweight births fluctuated from 5.0 to 5.7% (R^2^ = 0.79, *p* ≤ 0.01) and the national average rate of stillbirths fluctuated from 3.5 to 4.9% (R^2^ = 0.71, *p* ≤ 0.01). It was not possible to analyse these national average rates according to individual hospitals or broken out by GDM (Fig. 3).

## Discussion

This comprehensive audit of all maternity units in the Republic of Ireland found that the overall prevalence of GDM nationally increased from 3.1% in 2007 to 14.8% in 2016 (*p* < 0.01). The rate of increase gathered momentum after 2011 following the publication of a national guideline recommending the adoption of a more sensitive OGTT for selective screening. There was wide variation in the prevalence between maternity hospitals in 2007, which increased further over the decade. This may be explained by differences in pre-analytical laboratory standards and delays in implementing the national guidelines [22, 24]. Adoption of the guideline with full adherence in all units is likely therefore to further increase the percentage of women diagnosed with GDM nationally.

While the incidence of high birthweight babies ≥ 4.5 kg decreased over the study period, we were unable to show that this was associated with the increase in the diagnosis of GDM. Nor could the increase in women diagnosed be explained by an increase in maternal age nationally over the decade.

The implementation of the new guideline recommendations has resource implications. The pre-analytical glucose sample handling recommendations mean higher mean glucose concentrations and increased detection of GDM compared with existing hospital practices. More women will require surveillance in the third trimester with laboratory monitoring of plasma glucose and sonographic evaluation of fetal growth. An increase in women diagnosed with GDM also means that the number of women who require postnatal GDM testing to rule out Type 2 diabetes mellitus will increase.

One of the key strengths of the study is that it was based on data for over 99.7% of women who delivered babies in Ireland from 2008 to 2017. This included women attending all maternity hospitals/units receiving both public and privately-funded care. A further strength of the study was that we combined information from different datasets.

A limitation of the study was the restricted validity of the HIPE data. They are primarily administrative data and are not collected specifically for research purposes. For example, numbers of diagnoses are based on discharges rather than individual patients. Moreover, the data were based on amalgamated hospital reports and we were unable to capture details of women’s pregnancy circumstances or medical background. The data do not provide information on the level of adherence in each unit to screening based on risk factors. Our study did not examine patient-level data. The analysis was conducted at the national level only. We could not control for individual confounders such as maternal Body Mass Index, height, smoking status, alcohol consumption, previous history of GDM, family history of diabetes mellitus, ethnicity, gestational age at the time of the index OGTT, infant gender, parity, neonatal hypoglycemia or Neonatal Intensive Care admissions.

Following the introduction of the new IADPSG criteria in 2010, there has been a plethora of data looking at local prevalence of GDM [3–8]. Comparisons of reported prevalence rates for GDM from various countries using the IADPSG criteria in comparison to the criteria previously applied in those countries have shown variance from 3.5% (Western Australia) to 45.3% (United Arab Emirates) [7].

Comparing GDM prevalence between hospitals is confounded by differing diagnostic criteria due, in part, to differences in timing of adoption and adherence to the new national guidelines in the different maternity units. A survey conducted in 2012 found, for example, while all units screened women for GDM, 15 units followed the new criteria and used the recommended 75 g OGTT (79%), three units used a 100 g OGTT (16%) and one unit used a 50 g glucose challenge test [22]. A further national survey in 2015 of pre-analytical handling of oral glucose tolerance tests in pregnancy found that all 19 units were screening selectively [24]. Of the 19 units, 18 were using a one-step 75 g OGTT but four of the 18 were using a modified form. There were also variations in the pre-analytical laboratory standards. Our research group has previously shown that suboptimal pre-analytical handling of maternal plasma glucose samples results in underdiagnosis of GDM, particularly in obese women [24].

One of the factors that may increase the risk of developing GDM is advancing maternal age. Ireland has one of the highest proportion of women giving birth in their 40s. Over the decade, the average age of women giving birth (irrespective of parity) increased from 30.6 years in 2008 to 32.2 years in 2016 [23]. In 2016, more than a quarter of all births in Ireland was to women aged 35 or over [23]. Our findings suggest that the increase in older mothers may be contributing to the escalating rate of GDM. Another factor may be an increase in the incidence of maternal obesity, which is a major risk for GDM [25]. While this has been reported elsewhere for our own hospital, the information on national trends in BMI is not available.

Recent studies have suggested that GDM is associated with an increase in obstetric interventions [17]. Our findings show that the absolute number of women nationally with GDM who are having labor induced or are being delivered by CS rates has increased over the last decade. However, induction rates and CS rates have increased generally in the country [20, 26, 27]. Earlier interventions in pregnancy and increased rates of multiple pregnancies may have contributed to the modest 0.5% fall in the incidence of babies weighing ≥ 4.5kg rather than the major increase in women being treated for hyperglycemia [20]. The importance of confounding variables in perinatal trends highlights the importance of high quality intervention studies in evaluating the costs and benefits of different criteria for diagnosing GDM.

In a large USA study of over 125 million pregnancies in 1979–2010, 2.7% overall of cases hospitalised were diagnosed with GDM [28]. By 2008–10, the rate had increased to 5.8%. Extrapolating from population data, like our study the increased rate was associated with an increase in the prevalence of advanced maternal age and was associated with a decline in high birthweight. However, trends in adherence to testing were not known and patient level data on maternal obesity, smoking, ethnicity were not available. Also, the study was completed as the new post HAPO criteria were being developed and endorsed.

In conclusion, our study found that the introduction of the more sensitive 75 g OGTT in Irish maternity services was associated with a major increase in the prevalence of GDM nationally, with repercussions for healthcare resources. We identified persistent wide variations in GDM rates across the 19 hospitals, which may be explained by differences in the timing of and adherence to implementing national guidelines. Further research is required to determine the diagnostic criteria that optimise both short term and long term fetomaternal clinical outcomes [29]. Further research is also required to understand the varying patterns of guideline implementation across all maternity hospitals.

## Data Availability

The HIPE and NPRS data used in this national study were available to the researchers, subject to permission from the Healthcare Pricing Office, Health Service Executive. The data comply with national data requirements according to the Data Protection Acts 1998 to 2018 and Regulation (EU) 2016/679 of the European Parliament and the Council of 27 April 2016 also known as the General Data Protection Regulation.

## Abbreviations

GDM: Gestational diabetes mellitus
OGTT: Oral Glucose Tolerance Test
HAPO: Hyperglycemia and Adverse Pregnancy Outcomes
IADPSG: International Association of the Diabetes and Pregnancy Study Groups
ADA: American Diabetes Association
ACOG: American College of Obstetricians and Gynecologists
WHO: World Health Organization
FPG: Fasting Plasma Glucose
IOL: Induction of labor
CS: Cesarean section
LGA: Large for gestational age
HIPE: Hospital In-Patient Enquiry
ICD-10-AM: International Statistical Classification of Diseases and Related Health Problems, Tenth Revision, Australian Modification
HPO: Healthcare Pricing Office
NPRS: National Perinatal Reporting System
IMIS: Irish Maternity Indicator System
BNF01: Birth Notification Form
SPSS: Statistical Programme for Social Sciences
